# Health Literacy-related Safety Events: One Children’s Hospital’s Experience

**DOI:** 10.1097/pq9.0000000000000262

**Published:** 2020-04-22

**Authors:** Cori A. Gibson, Andrea K. Morrison, Michael F. Gutzeit

**Affiliations:** From the Quality Department,* Children’s Hospital of Wisconsin, Milwaukee, Wis.; † Department of Pediatrics, Medical College of Wisconsin Rita Higgins, RN, Children’s Hospital of Wisconsin, Milwaukee, Wis.

## Background:

Communication gaps leading to misunderstandings in medications, care plans, or health information can compromise patient safety. Focusing on health literate communication improves patient outcomes; however, many gaps in communication exist across healthcare. It is not known what deficits in health literate communication lead to patient safety events.

## Objectives:

Our aims were to (1) describe health literate communication themes identified in patient safety events and (2) identify health literacy-related areas for improving patient safety.

## Methods:

The safety events entered into a system-wide self-reported safety event collection database were prospectively tagged for health literacy events by a safety specialist trained in health literacy. The database was retrospectively queried for all health literacy tagged events in 9 months (September 2017–May 2018). The authors reviewed and independently coded health literacy-associated safety events. Qualitative content analysis of events facilitated by NVivo was completed to identify the health literacy-related safety event themes.

## Results:

Health literacy events comprised 4% (156/3,911) of self-reported safety events during the 9 months. Main themes of the health literacy safety events related to (1) medication; (2) discharge/transition; and (3) other health system events (Table 1). Subthemes of each of the events further described the event types. Health literacy-associated safety events were connected to all types of safety event outcomes (near miss, precursor, and serious safety events).

**Table 1.**
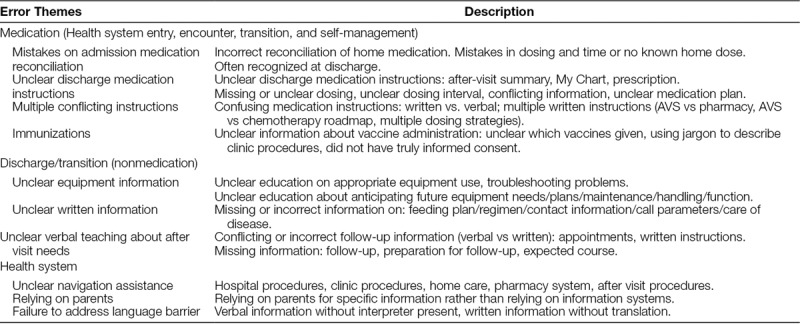
Health Literacy-related Safety Event Themes

## Conclusion/Implications:

Health literacy-related safety events occur in the healthcare environment and impact all types of safety event outcomes. Lack of health-literate practices impacts patient safety for patient/parent understanding of: (1) medication; (2) discharge/transition; and (3) health systems issues. Though health literacy-related safety events comprise a small number of overall safety events reported, this is likely an underestimate due to the self-reported nature of events, especially those types of events that a parent or staff has to recognize as a communication failure. Future work to improve patient safety outcomes can draw from these events and use health literacy-related interventions to improve communication, especially around medications, verbal communication, and transitions of care.

